# Observation of edge solitons in photonic graphene

**DOI:** 10.1038/s41467-020-15635-9

**Published:** 2020-04-20

**Authors:** Zhaoyang Zhang, Rong Wang, Yiqi Zhang, Yaroslav V. Kartashov, Feng Li, Hua Zhong, Hua Guan, Kelin Gao, Fuli Li, Yanpeng Zhang, Min Xiao

**Affiliations:** 1 Key Laboratory for Physical Electronics and Devices of the Ministry of Education & Shaanxi Key Lab of Information Photonic Technique, School of Electronic Science and Engineering, Faculty of Electronic and Information Engineering, Xi’an Jiaotong University, Xi’an, 710049 China; 2 Department of Applied Physics, School of Science, Xi’an Jiaotong University, Xi’an, 710049 China; 3 Institute of Spectroscopy, Russian Academy of Sciences, Troitsk, Moscow, 108840 Russia; 4 Wuhan Institute of Physics and Mathematics, Chinese Academy of Sciences, Wuhan, 430071 China; 5 Department of Physics, University of Arkansas, Fayetteville, AR 72701 USA; 6 National Laboratory of Solid State Microstructures and School of Physics, Nanjing University, Nanjing, 210093 China

**Keywords:** Atom optics, Solitons

## Abstract

Edge states emerge in diverse areas of science, offering promising opportunities for the development of future electronic or optoelectronic devices, sound and light propagation control in acoustics and photonics. Previous experiments on edge states in photonics were carried out mostly in linear regimes, but the current belief is that nonlinearity introduces more striking features into physics of edge states, leading to the formation of edge solitons, optical isolation, making possible stable lasing in such states, to name a few. Here we report the observation of edge solitons at the zigzag edge of a reconfigurable photonic graphene lattice created via the effect of electromagnetically induced transparency in an atomic vapor cell with controllable nonlinearity. To obtain edge solitons, Raman gain is introduced to compensate strong absorption experienced by the edge state during propagation. Our observations may open the way for future experimental exploration of topological photonics on this nonlinear, reconfigurable platform.

## Abstract (web summary)

Edge states are excitations existing at the boundary of truncated periodic materials with specific spectral degeneracies, and their properties are enriched when materials possess a nonlinear response. Here, the authors provide experimental evidence of edge soliton formation in a nonlinear photonic graphene lattice induced in an atomic vapour cell.

## Introduction

Edge states offer an efficient avenue for manipulation of the behavior of classical waves in engineered materials and play the important role in the design of desired optoelectronic devices^1–5^ that demands dynamic tunability^6^. One feasible way to achieve tunable devices is adopting nonlinearity that can be easily introduced into photonic systems^7–11^, in contrast to electronic ones. This advantage has stimulated investigations on nonlinear edge states, both topological and nontopological ones, in various structures, including photonic graphene^12,13^, where such effects as modulational instability^3,14^, solitons^3,4,15,16^, optical isolation^5^, and bistability^17^ were predicted that do not occur in pure electronic systems. Despite common expectations that nonlinear effects open fascinating prospects for control and manipulation of the edge states, the experimental demonstration of nonlinear edge states and edge solitons was not accomplished until now on photonic platforms.

On the other hand, recently introduced electromagnetically induced photonic lattices based on electromagnetically induced transparency (EIT)^18^ in multilevel atomic systems can mold the flow of light in a periodic manner and, in particular, allow induction of photonic graphene structures^19–23^. Based on the tunable atomic coherence, the absorption, dispersion, Raman gain^24^, and nonlinearity can all be easily controlled in such coherent atomic media^25–28^. The profiles of such lattices can be reconfigured dynamically^20^, so that edge states can be created or destroyed in them on demand. As to nonlinearity, its amplitude and nature can be easily changed by adjusting the laser frequency detuning under EIT conditions^25,26^. Therefore, coherently prepared atomic medium provides an ideal and powerful platform for the exploration of the edge states in strongly nonlinear regime.

In this article, by taking advantages of the controllable linear and nonlinear susceptibilities in an EIT medium, we experimentally demonstrate the formation and investigate propagation dynamics of the edge solitons in a reconfigurable photonic graphene constructed in an atomic vapor cell. The observation of edge solitons and such compact nonlinear edge excitations exhibit great significance. First, in contrast to localized linear edge states, edge solitons can travel along the edge over considerable distances without broadening and without noticeable radiation into the bulk, despite the fact that nonlinearity of the system tends to couple different modes. This ability to maintain localized shapes and peak intensity is supposed to be central for design of edge-state-based switching and routing devices. Second, nonlinear edge solitons, being authentic two-dimensional structures, are hybrids localized due to two different physical mechanisms. Their confinement in the direction perpendicular to the interface is inherited from the linear edge state from which edge solitons bifurcate with increase of the peak power, i.e., this confinement is of geometrical origin and it requires the lattice with specific symmetry, spectral properties, and proper truncation allowing existence of the linear edge states. In contrast, the confinement in the direction along the interface is due to nonlinear self-action in periodic refractive index landscape. The current work provides the effective illustration of the existence of such hybrid localization mechanisms and will certainly open broad prospects for investigation of interactions of nonlinear edge states. Third, the experimental system adopted here dramatically differs from conventional atomic lattices generally established with ultracold atoms and using very complicated laser cooling setups. Our edge solitons are formed in photonic lattices induced optically in a thermal atomic vapor cell that can be much closer to practical applications.

## Results

### Scheme for excitation of the edge states based on EIT

**Fig. 1:**
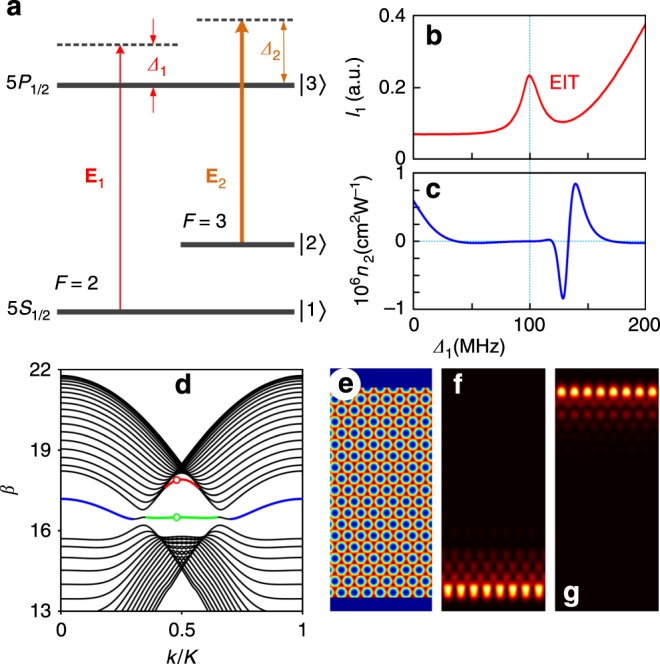
Atomic energy levels, linear bandgap structure, and edge states. **a** The driven ^85^Rb atomic transitions involve two hyperfine states $F = 2$ (level $|1\rangle$) and $F = 3$ ($|2\rangle$) of the ground state $5{\mathrm{S}}_{1/2}$ and one excited state $5{\mathrm{P}}_{1/2}$ ($|3\rangle$). ${\mathbf{E}}_1$ and ${\mathbf{E}}_2$ are the probe and the coupling fields, respectively. **b** The transmission spectrum for the probe beam. **c** Calculated nonlinear coefficient $n_2$ versus probe detuning $\Delta _1$. **d** Band structure of the photonic graphene in an EIT window calculated for $\Delta _1 = 135\,{\mathrm{MHz}}$ and $\Delta _2 = 100\,{\mathrm{MHz}}$.$\beta$ is the propagation constant, and $k$ is the Bloch momentum normalized to the width ${\mit{K}}$ of the first Brillouin zone. **e** Simulated honeycomb lattice with zigzag-bearded edges in the $y$ direction. The structure is periodic in the *x* direction, and we show six periods of the structure. **f** Simulated unconventional edge state located on the bearded edge corresponding to the red circle in (**d**). **g** Simulated edge state on the zigzag edge corresponding to the green circle in (**d**).

To demonstrate the formation of edge solitons in reconfigurable photonic graphene lattice, we employ the EIT effect. In our experiment, the probe field ${\mathbf{E}}_1$ (frequency $\omega _1$) co-propagates with coupling field ${\mathbf{E}}_2$ ($\omega _2$) along the *z* direction of the atomic cell to drive a three-level ${\mathrm{\Lambda }}$-type ^85^Rb atomic configuration schematically shown in Fig. 1a. The coupling field ${\mathbf{E}}_2$ possesses a hexagonal structure in the $(x,y)$ plane created by the interference of three tilted beams derived from the same diode laser. The propagation dynamics of the probe field ${\mathbf{E}}_1$ is defined by the susceptibility, $\chi = \chi ^{(1)} + 3\chi ^{(3)}\left| \psi \right|^2$^25^, where $\chi ^{(1)}$ and $\chi ^{(3)}$ are the linear and third-order susceptibilities, and $\psi$ is the envelope of the probe field ${\mathbf{E}}_1$. For appropriate detuning values $\Delta _1-\Delta _2=0$, the EIT window appears in the transmission spectrum of the probe field ${\mathbf{E}}_1$ (Fig. 1b). The magnitude and sign of the nonlinear coefficient $n_2 = 12\pi ^2\chi ^{(3)}/n_0^2c$^25^ (here $n_0= 1$ is the background refractive index) within the EIT window can be easily controlled by the detuning of the probe beam $\Delta _1$ [(Fig. 1c) and “Methods”]. Since for $|{\mathbf{E}}_1| \ll |{\mathbf{E}}_2|$ the linear susceptibility *χ*^(1)^~|Ω_2_|^−2^, where $\Omega _2$ is the Rabi frequency of the coupling field ${\mathbf{E}}_2$, the hexagonal |Ω_2_|^2^ distribution, when inverted, creates a honeycomb lattice for the probe field^19^—the photonic analogue of graphene lattice. Proper truncation of such a lattice (for example by an adjustable rectangular slit) creates a ribbon, periodic in $x$ and having zigzag-bearded boundaries in $y$, whose theoretical refractive index profile $[1 + \chi ^{\left( 1 \right)}]^{1/2}$ is shown in Fig. 1e. Linear modes of such a ribbon are Bloch waves $\psi = w\left( {x,y} \right)e^{{\mathrm{i}}\beta z + {\mathrm{i}}kx}$ (see “Methods” for dimensionless equation governing probe-field propagation and ref. ^15^), where $w$ is periodic in $x$ with lattice period $X$ and localized in $y$, $\beta$ is the dimensionless propagation constant, and $k$ is the Bloch momentum along the *x*-axis. The spectrum $\beta (k)$ (Fig. 1d) reveals the formation of linear edge states at bearded (red and blue curves, Fig. 1f) and zigzag (green curve, Fig. 1g) boundaries, while black curves correspond to bulk modes. These edge states are of geometrical origin: they only form for proper truncation of the lattice with specific degeneracies (Dirac points in our case) in the spectrum (in tight-binding models they are known as zero-energy edge states^21^). Edge-state localization in $y$ is controlled by $k$.

**Fig. 2:**
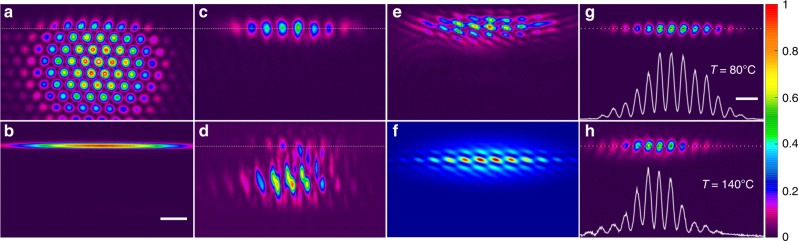
Experimental demonstration of the edge state at the zigzag edge. **a** Interference pattern of three coupling beams creating the lattice with a lattice constant of $112\,\upmu {\mathrm{m}}$. This hexagonal lattice induces the honeycomb lattice in Fig. 1e under EIT conditions. The edge marked by the dotted line corresponds to zigzag edge. **b** The incident stripe probe beam. Scale bar: 200 μm. **c** The edge state excited by the probe beam at $\Delta _1 = 135\,{\mathrm{MHz}}$ with the probe power being $100\,\upmu {\mathrm{W}}$. **d** Diffraction of the probe beam into the bulk of the lattice at $\Delta _1 = 105\,{\mathrm{MHz}}$. **e** Interference pattern of the output probe beam from panel **c** with a reference beam illustrating staggered phase of the edge state. **a**–**e** share the same scale bar defined in (**b**). **f** Theoretical interference pattern calculated for extended linear edge state. **g**, **h** Output probe beams for different temperatures (effectively corresponding to different propagation distances) at $\Delta _1 = 135\,{\mathrm{MHz}}$ revealing motion of the edge state. White lines at the bottom show intensity profiles of the probe beam along the dashed lines. Considering the absorptive nature of atomic medium, the linear gain $g$ (which only affects the visibility, but not the profile of the beams) of the CCD camera is used to improve the appearance of figures. The CCD gain for (**g**) and (**h**) are $g = 0$ (no gain) and $g = 8$, respectively. Scale bar: 200 μm.

**Fig. 3:**
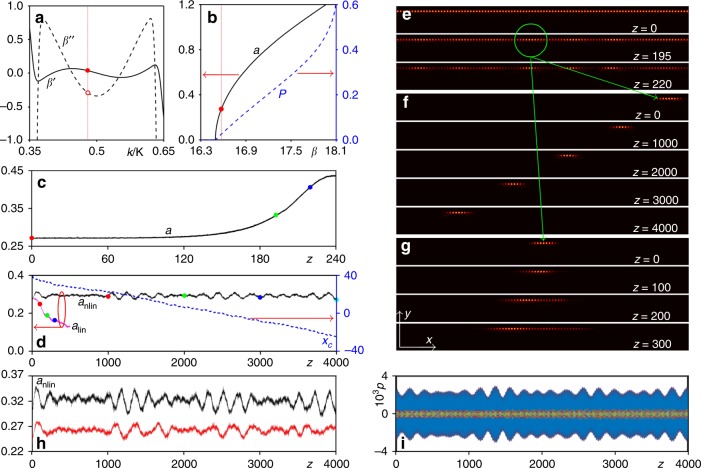
Numerical simulations of edge quasi-solitons. **a** First $\beta^ {\prime}$ and second-order $\beta^ {\prime\prime}$ derivatives for green branch of edge states from Fig. 1c. Vertical dotted line indicates the Bloch momentum $k = 0.48\,{\mathit{K}}$. **b** Nonlinear edge state family at $k = 0.48\,{\mathit{K}}$. Solid and dashed curves show peak amplitude $a$ and norm $P$ versus $\beta$. **c** Peak amplitude of the nonlinear edge state with $\beta = 16.573$ (corresponding to the red dot in (**b**)) versus distance illustrating the development of modulation instability. **d** Amplitude $a_{{\mathrm{nlin}}}$ and center position $x_c$ of quasi-soliton from panel **f** versus propagation distance. The amplitude $a_{{\mathrm{lin}}}$ for the same input in linear medium is shown too. **e** Nonlinear edge state intensity distributions at different propagating distances corresponding to the dots in (**c**). **f** Quasi-soliton intensity distributions at different propagation distances corresponding to the dots in $a_{{\mathrm{nlin}}}$ curve in (**e**). **g** Diffraction in linear medium, distributions shown correspond to the dots in $a_{{\mathrm{lin}}}$ curve in (**d**). **h** Evolution of the peak amplitude $a_{{\mathrm{nlin}}}$ of the quasi-soliton in the case when input amplitude was increased (top curve) and decreased (bottom curve) by $10{\mathrm{\% }}$. **i** Projection $p$ of the soliton field distribution at different distances on bulk Bloch modes of the system.

Experimentally created lattice (${\mathbf{E}}_2$ field) was truncated with a slit to form zigzag boundary (dotted line) in honeycomb refractive index distribution as shown in Fig. 2a (see also “Methods”). By properly setting the incident angle $\alpha$ of the stripe probe beam (Fig. 2b) along the boundary to match the momentum of the edge state from the range of $K/3 \le k \le 2K/3$ [Fig. 1(d)], where $K = 2\pi /X$ is the width of the Brillouin zone, one can achieve efficient edge-state excitation. The depth of our lattice can be easily changed by changing frequency detuning, so first we illustrate the creation and destruction of the edge states by varying $\Delta _1$, while keeping $\Delta _2 = 100\,{\mathrm{MHz}}$ fixed. We achieve efficient excitation of the edge state (Fig. 2c) at $\Delta _1 = 135\,{\mathrm{MHz}}$ (corresponding susceptibility created by the coupling beam is $\chi ^{(1)}\sim 3.4 \times 10^{ - 4}$) at the angle of incidence $\alpha \approx 0.8^\circ$. The interference between the output probe and reference beam (derived from the same laser as the probe beam) reveals staggered phase distribution along the *x*-axis in probe beam (Fig. 2e) in agreement with the numerical results (Fig. 2f), which is a signature of the edge-state formation^21^. In contrast, when detuning is set to $\Delta _1 = 105\,{\mathrm{MHz}}$ for the same angle of incidence *α*, the induced susceptibility $\chi ^{\left( 1 \right)}\sim 4.5 \times 10^{ - 5}$ is insufficient to support edge state formation on the cell length $(7.5\,{\mathrm{cm}})$ and one observes diffraction into the bulk (see Fig. 2d; Supplementary Fig. 1). This illustrates suitability of the setting for all-optical manipulation of the edge states. Another advantage of the system is that the increase of the atomic density (controlled by the temperature of the atomic ensemble) is effectively translated into the increase of propagation path of the probe beam in the lattice^29^. Thus, increasing temperature of the medium from $80^{\mathrm{o}}{\mathrm{C}}$ to $140^{\mathrm{o}}{\mathrm{C}}$ at $\Delta _1 = 135\,{\mathrm{MHz}}$ allows us to detect clear displacement of the edge state along the zigzag boundary (Fig. 2g, h) due to its small, but nonzero group velocity $\beta^ {\prime} = d\beta /dk$ (see green state in Fig. 1c and the corresponding $\beta^ {\prime}(k)$ curve in Fig. 3a). Notice that the probe beam gets attenuated even within the EIT window, since this effect only suppresses rather than completely eliminates the absorption, so at higher temperatures (resulting in stronger absorption), we had to adjust the CCD camera gain $g$ as indicated in experimental images, where it is appropriate ($g = 0$ means no gain).

### Properties of the edge solitons

As numerical simulations (conducted in ideal lossless case) show, the formation of edge solitons is tightly connected with the phenomenon of modulation instability (MI) of periodic nonlinear edge states that for positive $n_2$ can only occur in the range of momentum $k$ values that meet $\beta^ {\prime\prime} = d^2\beta /dk^2 \, < \, 0$ (Fig. 3a) (see ref. ^15^). Representative theoretical family of nonlinear edge states at $k = 0.48\,{\mathrm{K}}$ (dotted line in Fig. 3a) is shown in Fig. 3b (“Methods“). Nonlinear edge state bifurcates from linear one with increase of propagation constant $\beta$: its peak amplitude $a = {\mathrm{max}}\left| \psi \right|$ and norm per $x$-period $P = \int_{ - \infty }^{ + \infty } dy\int_{ - X/2}^{ + X/2} \left| \psi \right|^2dx$ increase away from bifurcation point (Fig. 3b). Notice that the ribbon is finite in the $y$ direction, so the integration in $y$ in definition of power can be performed just over the region well exceeding the $y$-width of the ribbon. For a given $k$, we consider only nonlinear edge states in the gap $\left( {\beta \le 18.1} \right)$ to prevent coupling with bulk modes. We choose a slightly perturbed nonlinear edge state at $\beta = 16.573$ (dotted line in Fig. 3b) as an input in Fig. 3e to check its propagation (this can always be done, since $\beta$ parameterizes the family of the nonlinear edge states). The dependence $a\left( z \right)$ (Fig. 3c) and breakup of the state into sets of bright spots (precursors to quasi-solitons) upon propagation (Fig. 3e) clearly indicate the development of MI of nonlinear edge state. We then isolate one bright spot from MI pattern marked with green circle in Fig. 3e [the MI pattern was taken at the distance $z = 195$ and it corresponds to the green dot in Fig. 3c) and let it propagate in nonlinear (Fig. 3f) and linear (Fig. 3g) media. In other words, Fig. 3f, g show propagation of the same input state in the EIT system with the third-order Kerr nonlinearity switched on and off, respectively. In the former case, one clearly observes the formation of slowly moving quasi-soliton (akin to solitons predicted in topological systems^15^), whose velocity is determined by the group velocity *β*′ of the linear edge state on which soliton is constructed, and whose peak amplitude $a_{{\mathrm{nlin}}}\left( z \right)$ only slightly oscillates, while center $x_c$ changes linearly with $z$ (Fig. 3d). Intensity distributions at different distances corresponding to dots in $a_{{\mathrm{nlin}}}\left( z \right)$ dependence confirm invariable shape over distances greatly exceeding cell length and the absence of radiation into the bulk. In contrast, in the linear case the same input rapidly spreads along the boundary, while corresponding peak amplitude $a_{{\mathrm{lin}}}\left( z \right)$ decreases (Fig. 3d). Notice that even though edge solitons obtained here are not topological, they are robust entities. In the presence of localized edge defects in the form of missing channel, they typically bounce back and keep moving along the edge in the opposite direction (see Supplementary Figs. 8 and 9 and Supplementary Note 6). Their amplitude and width remain practically unchanged after collision with a defect, indicating on the possibility of practically 100% defect-mediated coupling of power into state moving in the opposite direction along the edge, without radiative losses into bulk. The robustness of the edge solitons was also verified by propagating them in the presence of initial random perturbations, or by increasing or decreasing the input soliton amplitude by $10{\mathrm{\% }}$, as displayed in Fig. 3h. One finds that even such considerable perturbation leads to small oscillations in soliton amplitude that self-adjusts quickly to new input power level. In addition, to prove the absence of resonant scattering into linear bulk modes, we calculated radiative losses from moving edge soliton during propagation by projecting field distribution at different distances on all bulk Bloch modes of the system: $p = \langle\psi _{{\mathrm{soliton}}},\psi _{{\mathrm{bulk}}}\rangle/\langle\psi _{{\mathrm{bulk}}},\psi _{{\mathrm{bulk}}}\rangle$, as shown in Fig. 3i. The projections remain negligibly small over all distances (<$0.3{\mathrm{\% }}$), which means that the efficiency of excitation of the bulk modes is extremely low, it is nonresonant, and cannot lead to soliton decay.

### Experimental observation of the edge solitons

**Fig. 4:**
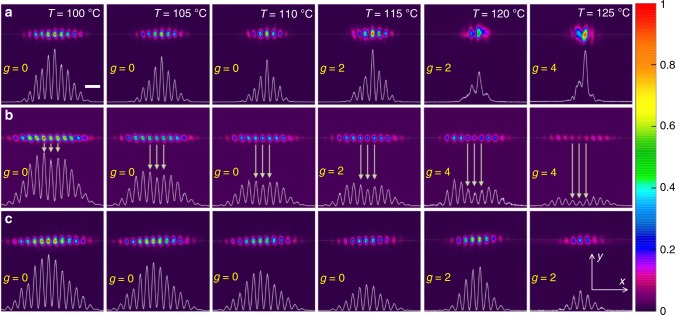
Focusing, defocusing, and diffraction-free propagation of the nonlinear edge states. **a** Self-focusing of the edge state at $n_2 \, > \, 0$ (Δ_1_ = 140 MHz) with increase of temperature. Scale bar: 200 μm. **b** Self-defocusing of the edge state at $n_2 \, < \, 0$ (Δ_1_ = 125 MHz) with increase of the temperature. **c** Formation of diffraction-free edge states under the action of focusing nonlinearity $n_2 \, > \, 0$ (Δ_1_ = 135 MHz) weaker than in panel **a**, when the pattern keeps the same functional form for different temperatures. The probe power is $400\,\upmu {\mathrm{W}}$. **b**, **c** share the same scale bar as (**a**).

In experiment, once the system is tuned into the regime with focusing or defocusing nonlinearity by adjusting $\Delta _1$ (Fig. 1c), one can observe considerable nonlinearity-induced reshaping of the edge states that is enhanced at higher temperatures (Fig. 4). In addition to two-dimensional intensity distributions, at the bottom of each panel we show one-dimensional profiles at the boundary along the dotted white lines. First row of Fig. 4a illustrates clear self-focusing of the edge state down to several lattice periods with increasing temperature for $n_2 \, > \, 0$ (Δ_1_ = 140 MHz). Figure 4b illustrates self-defocusing of the edge state in the region $n_2 \, < \, 0$ (Δ_1_ = 125 MHz) and appearance of the dip marked by the arrows in the beam profile that becomes more pronounced at higher temperatures. When $\Delta _1$ is set such as to make $n_2$ slightly exceeding 0, the edge states also experience diffraction, which is somewhat slower than diffraction in the case of $n_2 \, < \, 0$ (see Supplementary Fig. 7), because nonlinearity for small positive $n_2$ only partially compensates diffractive broadening. Finally we set (Δ_1_ = 135 MHz) that corresponds to strong focusing nonlinearity $n_2 \, > \, 0$ to compensate the beam diffraction, so that the confined edge state experiences neither noticeable defocusing nor focusing with increasing temperature (the ratio of amplitudes of different peaks remain basically the same) as shown in Fig. 4c, which illustrates the nonlinear behavior with apparent formation of soliton-like profiles and their dynamical self-adjustment with account of power losses for a wide range of temperatures. We attribute this important regime to dynamical balance between diffraction in the lattice and nonlinear self-action, which would provide the proper condition for the potential formation of edge solitons. Even though attenuation is unavoidable in our setting (in the absence of Raman gain), the total power losses upon propagation over atomic cell are not very high, and the nonlinear edge states excited with sufficiently high input powers can still dynamically accommodate their shapes in accordance with slowly decaying power without entering the regime of linear diffraction (at least on the atomic cell length). The patterns shown in Fig. 4c should therefore be treated as such dynamical self-sustained states (albeit they are not exact conservative solitons due to the presence of losses) slowly self-adjusting with $z$ in accordance with gradually decreasing peak amplitude. Clear transition between linear diffraction and formation of such self-sustained nonlinear states can be also achieved by increasing input power at fixed temperature and detuning, as shown in Supplementary Fig. 10.

**Fig. 5:**
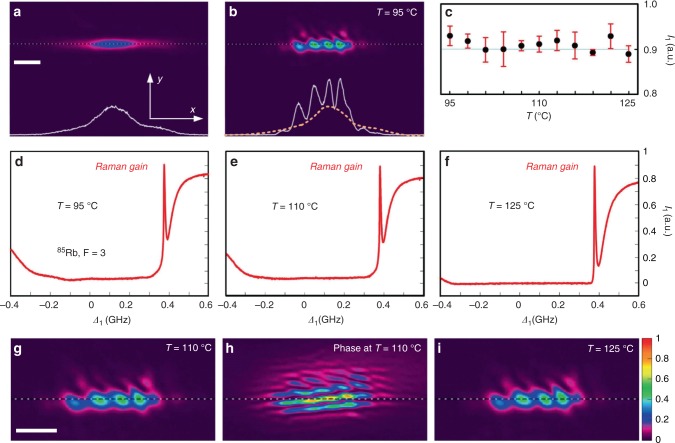
Formation of edge solitons in the presence of Raman gain. **a** Input probe beam. Scale bar: 200 μm. **b** Output probe beam in the presence of Raman gain at $T = 95^{\mathrm{o}}{\mathrm{C}}$. The cross-section of the incident beam shown in both panels **a** and **b** enables comparison with the output profile that confirms suppression of losses by Raman gain. **a**, **b** share the same scale bar. **c** Output probe intensity with Raman gain versus different temperatures (atomic density). Each black dot represents the peak intensity of the probe beam with $\Delta _1 = 380\,{\mathrm{MHz}}$, where the Raman gain locates. The error bars indicate the standard deviation for three measurements. **d**–**f** Output probe spectra versus $\Delta _1$. The peak heights correspond to the dots in (**c**). Profiles of the output probe beams in the presence of Raman gain at $T = 110^{\mathrm{o}}{\mathrm{C}}$ (**g**) and $T = 125^{\mathrm{o}}{\mathrm{C}}$ (**i**). Scale bar: 200 μm. **h** Interference pattern with reference beam corresponding to (**g**). **h**, **i** share the same scale bar as (**g**).

To demonstrate the formation of edge solitons under practically ideal loss-free conditions, we further add an extra Gaussian-profile pump field (see “Methods“) that provides a Raman gain for the probe field compensating intrinsic absorption^16^. Figure 5a, b shows, respectively, the incident stripe probe beam and the formed edge soliton at $T = 95^{\mathrm{o}}{\mathrm{C}}$ and $\Delta _1 = 380\,{\mathrm{MHz}}$, where the Raman gain peak lies (Fig. 5d–f). Dependence of the Raman gain peak ($\Delta _1 = 380\,{\mathrm{MHz}}$) on the temperature (Fig. 5c) demonstrates that the output distributions are very robust and practically not affected by temperature variation due to Raman gain. To illustrate Raman gain clearly, Fig. 5d–f displays probe spectra versus $\Delta _1$ at three different temperatures. One finds that the height of the Raman gain peak basically does not change even when the absorption grows (the notch deepens and the background gets close to 0) with temperature. The fact that Raman gain effectively balances the absorption and helps formation of edge solitons is further confirmed by comparison of very similar output patterns at $110^{\mathrm{o}}{\mathrm{C}}$ and $125^{\mathrm{o}}{\mathrm{C}}$ (Fig. 5g, i). The staggered phase (Fig. 5h) confirms that the edge state is excited.

## Discussion

In conclusion, we have experimentally demonstrated edge solitons in a photonic graphene lattice induced in a multilevel atomic system. This reconfigurable atomic system opens promising prospects for all-optical control of the formation and propagation of the edge states in different nonlinear regimes and for different lattice configurations. The possibility to implement Raman gain in the system allows to compensate for intrinsic losses, and study physics of nonlinear edge states under practically ideal loss-free conditions. Our work opens the door for experimental exploration of nonlinear dynamics of edge states not only in nontopological but also in various topological systems^3,4^, including those based on valley Hall effect^30^. Moreover, the current setting with Raman gain and loss is promising for exploration of non-Hermitian edge states and edge state lasers in two-dimensional geometries.

It is also worth mentioning that edge solitons reported here are qualitatively different from usual bulk and surface two-dimensional solitons. Due to different localization mechanisms involved, edge solitons do not feature power threshold for their existence. This is in complete contrast to conventional surface solitons^31–36^ whose most representative feature in both one- and two-dimensional settings is the existence of the power threshold required for their excitation—a manifestation of the fact that surface solitons do not bifurcate from linear localized modes and that it is only nonlinearity that leads to the confinement of such states in both transverse directions in the two-dimensional case. The same argument in two-dimensional settings applies to bulk lattice solitons bifurcating from delocalized Bloch waves, that still feature the same localization mechanism in two dimensions and also exist above energy flow threshold^37^. Besides the atomic medium adopted in this work, we believe that nonlinear two-dimensional generalizations of the edge states can be also potentially observed (even though they are not reported yet) in photorefractive crystals^38^.

## Methods

### Experimental setup

Our lattice is induced by three coupling beams (wavelength $\lambda _2 = 794.975\,{\mathrm{nm}}$, vertical polarization, 20 mW) derived from the same continuous-wave single-mode tunable external cavity diode laser (ECDL), that intersect in the center of the atomic vapor cell. These broad Gaussian coupling beams are symmetrically arranged with respect to the $z$ direction (with the same small angle of $\sim 0.4^{\mathrm{o}}$ between any two of them), inducing a hexagonal lattice in the $(x,y)$ plane. Due to small angle between the beams, the lattice pattern remains practically unchanged in the *z* direction over the distance of $10\,{\mathrm{cm}}$ that exceeds the length of $7.5\,{\mathrm{cm}}$ atomic cell. The lattice is truncated by using an adjustable rectangular slit (with a maximum opening window of $1\,{\mathrm{cm}}$) resulting in the formation of the structure with zigzag edge, as shown in Fig. 2a. The probe beam ${\mathbf{E}}_1$ ($\lambda _1 = 794.981\,{\mathrm{nm}}$, horizontal polarization) from another ECDL is transformed into a stripe beam ($0.4\,{\mathrm{mW}}$) by another adjustable rectangular slit, and its Fourier transform is imaged into the zigzag edge of the lattice. The $7.5\,{\mathrm{cm}}$ long cell is wrapped with *μ*-metal sheets and heated by a heat tape to control the temperature (and, therefore, the atomic density) of the medium. At the output of the cell, a polarization beam splitter (PBS) is applied to filter out the coupling field, so that only the probe field can reach the CCD camera. The phase of the output probe beam confined at the zigzag edge is measured by interfering it with a reference beam (introduced into the optical path via a $50/50$ beam splitter) from the same diode laser as the probe beam. To introduce Raman gain, a Gaussian-profile pump field (wavelength $\lambda _3 = 780.24\,{\mathrm{nm}}$, vertical polarization, $10\,{\mathrm{mW}}$) is injected into the atomic cell with the same direction as one of the coupling beams to drive a four-level N-type atomic configuration, see Supplementary Fig. 2 and Supplementary Note 2 for details.

### The dynamic propagation equation and susceptibilities in an EIT window

Propagation of light in the atomic vapor is described by the Schrödinger-like paraxial wave equation,

1 $${\mathrm{i}}\frac{\partial }{{\partial z}}\psi \left( {x,y,z} \right) = - \frac{1}{{2k_0}}\left( {\frac{{\partial ^2}}{{\partial x^2}} + \frac{{\partial ^2}}{{\partial y^2}}} \right)\psi \left( {x,y,z} \right) - \frac{{k_0}}{{n_0}}{\mathrm{\Delta }}n\left( {x,y} \right)\psi \left( {x,y,z} \right),$$

where $\psi$ is the envelope of the probe field ${\mathbf{E}}_1$, $z$ is the propagation distance, $k_0 = (2n_0\pi )/\lambda _1$ is the wavenumber in the medium, $n_0 = 1$ is the background refractive index, and ${\mathrm{\Delta }}n \approx \frac{1}{2}(\chi ^{\left( 1 \right)} + 3\chi ^{\left( 3 \right)}\left| \psi \right|^2)$ is the refractive index change that exhibits a honeycomb profile. Within EIT window the susceptibilities are given by $\chi ^{(1)} = {\mathrm{i}}{\it{N}}\left| {\mu _{31}} \right|^2\left( {\hbar {\it{\epsilon }}_0{\it{F}}} \right)^{ - 1}\left[ {1 - 2\gamma _{21}/\left( {2\gamma + \gamma _{31}} \right)} \right]$, with $F = (\gamma - {\mathrm{i}}\Delta _1) + \left| {{\mathrm{\Omega }}_2} \right|^2[\gamma _{21} - {\mathrm{i}}({\Delta}_1 - \Delta _2)]^{ - 1}$ and $\gamma = (\gamma _{21} + \gamma _{31} + \gamma _{32})/2$, and by $\chi ^{(3)} = - {\mathrm{i}}N\left| {\mu _{31}} \right|^2\left( {\hbar {\it{\epsilon }}_0F} \right)^{ - 1}\left[ { - \left| {{\Omega}_2} \right|^2/(2\gamma + \gamma _{31})} \right]\left[ {(F + F^ \ast )/|F|^2} \right]$. Here, $\Delta _1$ ($\Delta _2$) is the detuning between the resonant transition frequency $| 1 \rangle \to |3\rangle$ ($| 2 \rangle \to |3\rangle$) and the frequency of field ${\mathbf{E}}_1$ (${\mathbf{E}}_2$); ${\mathrm{\Omega }}_2 = \mu _{32}|{\mathbf{E}}_2|/\hbar$ is the Rabi frequency for the coupling field; $\mu _{mn}$ is the dipole momentum for transition $| m \rangle \to |n\rangle$; $\gamma _{31}$ and $\gamma _{32}$ are the spontaneous decay rates of the excited state $| 3 \rangle$ to the ground states $| 1 \rangle$ and $| 2 \rangle$, respectively; $\gamma _{21}$ is the nonradiative decay rate between two ground states; and $N$ is the atomic density at the ground state $| 1 \rangle$. By replacing $(x,y,z)$ with $(x/r_0,y/r_0,z/k_0r_0^2)$, the normalized governing equation can be written as

2 $${\mathrm{i}}\frac{\partial }{{\partial z}}\psi \left( {x,y,z} \right) = - \frac{1}{2}\left( {\frac{{\partial ^2}}{{\partial x^2}} + \frac{{\partial ^2}}{{\partial y^2}}} \right)\psi \left( {x,y,z} \right) - \frac{{k_0^2r_0^2}}{{n_0}}{\mathrm{\Delta }}n\left( {x,y} \right)\psi \left( {x,y,z} \right),$$

where $r_0$ is related to the probe width. When only linear susceptibility is considered, we solve the equation with the ansatz $\psi = w\left( {x,y} \right)e^{{\mathrm{i}}\beta z + {\mathrm{i}}kx}$ by adopting the plane-wave expansion method, and obtain the band structure (Fig. 1c) as well as linear edge states (Fig. 1e, f), see Supplementary Note 4 for details.

## Supplementary information

Supplementary Information

## Data Availability

The data that supports the results within this paper are available from the corresponding authors upon reasonable request.

## Code availability

The custom code used to obtain the results within this paper are available from the corresponding authors upon reasonable request.
