# Supplementary material for: Observation of edge solitons in photonic graphene
Source: Nat Commun. 2020 Apr 20;11:1902. doi: 10.1038/s41467-020-15635-9 (PMC7171198; doi:10.1038/s41467-020-15635-9)
Supplement: Supplementary file 1 — Supplementary Information [file 41467_2020_15635_MOESM1_ESM.pdf]

Supplementary Information

Observation of Edge Solitons in Photonic Graphene

Zhang et al.

Supplementary Note 1: Band structure for edge states at $\Delta_1 = 105$ MHz.

The depth of the induced lattice strongly depends on the detuning value. Thus, when $\Delta_1 = 105$ MHz the induced lattice is too shallow. The example of the band structure presented in Supplementary Fig. 1 shows that in this case edge states (green line) are not sufficiently gapped.

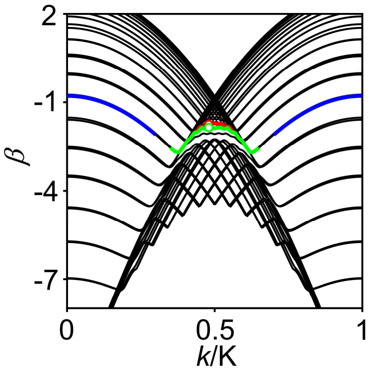

Supplementary Figure 1. The band structure at $\Delta_1 = 105$ MHz. Other parameters are same as those in Fig. 1(d).

Supplementary Note 2: Experimental arrangement for edge solitons with Raman gain

The experimental arrangement for generation of the edge solitons with Raman gain is shown in Supplementary Fig. 2(a). The EIT window is induced in the subsystem $|1\rangle \rightarrow |3\rangle \rightarrow |2\rangle$, in which we demonstrate the focusing/defocusing effects (Fig. 4 in the main text) of the excited edge wave, which experiences strong absorption during propagation. The description on the three-level subsystem is given in the **Methods** section.

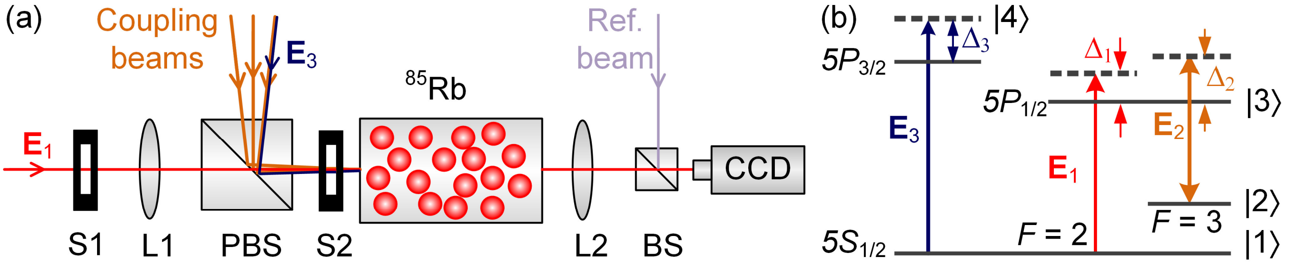

Supplementary Figure 2. (a) Beam arrangements for excitation of the edge solitons. (b) The four-level atomic configuration for generating Raman gain on the probe beam. $\Delta_i = \omega_{ij} - \omega_i$ is the detuning between the atomic resonant frequency ω_{ij} $(i,j = 1,2,3)$ and the laser frequency ω_i of $\mathbf{E}_i$ $(i = 1,2,3)$. $\mathbf{E}_1$: probe field (horizontal polarization); $\mathbf{E}_2$: Coupling field that is formed by interfering three coupling beams (vertical polarization); $\mathbf{E}_3$: pump beam (vertical polarization); S1: adjustable rectangular slit for making a stripe probe beam; L1: lens for imaging the stripe probe beam into the zigzag boundary of the coupling lattice; PBS: polarization beam splitter; S2: adjustable rectangular slit for cutting the zigzag boundary; L2: lens for imaging the output probe beam onto the CCD camera; BS: beam splitter for introducing the reference beam.

To form edge solitons, we add a Gaussian pump field $\mathbf{E}_3$ (wavelength $\lambda_3 = 780.2$ nm, frequency ω_3, vertical polarization, coupling the transition $|1\rangle \rightarrow |4\rangle$) to drive a four-level N-type atomic configuration [Supplementary Fig. 2(b)], which can provide an amplification for the probe beam to balance the intrinsic absorption of the atomic vapor. The vertically-polarized pump beam is injected into the medium along the

same direction as one of the coupling beams. Actually, the pump beam can modulate the imaginary part of the refractive index (i.e. make it negative that corresponds to gain) without affecting the real part of the refractive index under certain parametric mechanisms [1]. So the honeycomb lattice "written" by the coupling field certainly persists, when Raman gain is effectively introduced. As a consequence, the probe field with Raman gain can excite the edge state and form edge solitons by taking advantages of the double balance between dispersion and Kerr nonlinearity as well as gain and loss.

Supplementary Note 3: Atomic density versus the temperature

With the atomic density increased from N_1 to N_2, the equivalent optical path amounts to $N_2/N_1 \times L$, where L is the effective propagation length of probe beam at N_1. The relation between temperature T and atomic density is shown in Supplementary Fig. 3.

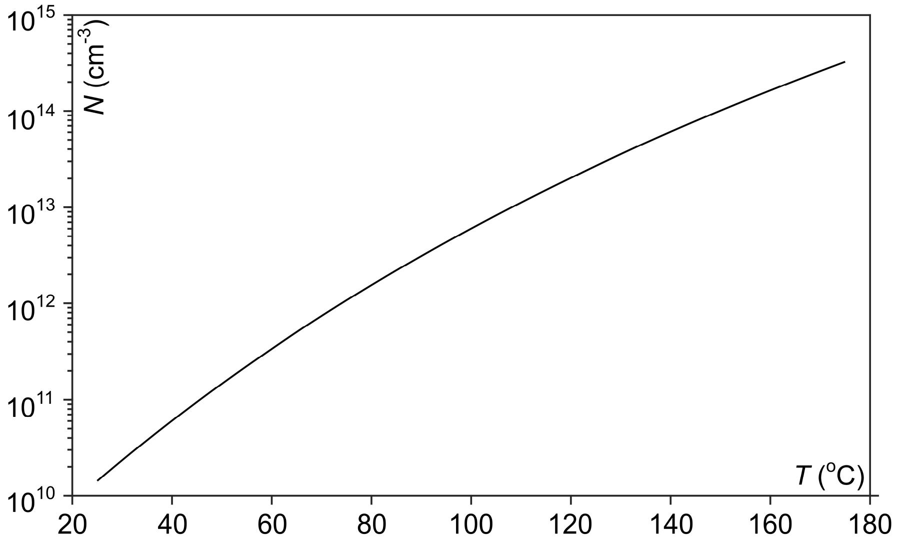

Supplementary Figure 3. The relationship between atomic density and temperature.

Supplementary Note 4: Theoretical model and numerical simulation details

Light propagation in the EIT atomic medium can be described by the Schrödinger-like paraxial wave equation

$$\mathrm{i}\frac{\partial}{\partial Z}\psi(X,Y,Z) = -\frac{1}{2k_0}\nabla^2\psi(X,Y,Z) - \frac{k_0}{n_0}\Delta n(X,Y)\psi(X,Y,Z), \qquad (1)$$

where ∇^2 is the Laplacian operator, Δn is the refractive index change, $k_0 = (2n_0\pi)/\lambda$ is the wavenumber, $n_0 = 1$ the background refractive index, and ψ is the electric envelope of the probe beam. If we set $x = X/r_0$, $y = Y/r_0$ and $z = Z/k_0 r_0^2$ with r_0 being the probe beam width, Supplementary Equation (1) can be transformed into a dimensionless equation, as

$$\mathrm{i}\frac{\partial}{\partial z}\psi(x,y,z) = -\frac{1}{2}\left(\frac{\partial^2}{\partial x^2} + \frac{\partial^2}{\partial y^2}\right)\psi(x,y,z) - \frac{k_0^2 r_0^2}{n_0}\Delta n(x,y)\psi(x,y,z), \qquad (2)$$

and the refractive index can be written as $\sqrt{1+\chi}$. In EIT atomic systems, the refractive index change is much smaller than 1 and can be directly written as $\Delta n \approx 0.5\chi$. If we consider the first-order and third-order susceptibilities in the system, the total susceptibility can be written as

$$\chi = \chi^{(1)} + 3\chi^{(3)}|\psi|^2, \tag{3}$$

with

$$\chi^{(1)} = \mathrm{i}\frac{N|\mu_{31}|^2}{\hbar\epsilon_0}\frac{1}{F}\left[1 - \frac{2\gamma_{21}}{2\gamma+\gamma_{31}}\right]$$

and

$$\chi^{(3)} = -\mathrm{i}\frac{N|\mu_{31}|^2}{\hbar\epsilon_0}\frac{1}{F}\left[-\frac{|\Omega_2|^2}{2\gamma+\gamma_{31}}\cdot\frac{F+F^*}{|F|^2}\right]$$

where $F = (\gamma - i\Delta_1) + |\Omega_2|^2[\gamma_{21} - i(\Delta_1 - \Delta_2)]^{-1}$ and $\gamma = (\gamma_{21} + \gamma_{31} + \gamma_{32})/2$. Here, Δ_1 (Δ_2) is the detuning between the resonant transition frequency $|1\rangle \to |3\rangle$ ($|2\rangle \to |3\rangle$) and the frequency of field $\mathbf{E}_1$ ($\mathbf{E}_2$); $\Omega_2 = \mu_{32}\ |E_2|/\hbar$ is the Rabi frequency for the coupling field; μ_{mn} is the dipole momentum for transition $|i\rangle \to |j\rangle$; γ_{31} and γ_{32} are the spontaneous decay rates of the excited state $|3\rangle$ to the ground states $|1\rangle$ and $|2\rangle$, respectively; γ_{21} is the nonradiative decay rate between two ground states; and N is the atomic density at the ground state $|1\rangle$.

In Supplementary Figs. 4(a,b), the profiles of the first- and third-order susceptibilities, which are honeycomb lattices with zigzag-bearded edges resulted from the three-beam interference method, are shown. The used parameters are given in the caption. The order of $\chi^{(1)}$ is 10^{-4} and that of $\chi^{(3)}$ is 10^{-12} $(\mathrm{mV}^{-1})^2$. Solution of Supplementary Equation (2) can be written as $\psi(x,y,z) = w(x,y)\exp(\mathrm{i}\beta z + \mathrm{i}ky)$ with $w(x,y)$ being the Bloch wave, β the energy and k the Bloch momentum. Plugging this solution into Supplementary Equation (2), one obtains

$$-w\beta \ = -\frac{1}{2}\left(\frac{\partial^2 w}{\partial x^2} + \frac{\partial^2 w}{\partial y^2} + 2\mathrm{i}k\frac{\partial w}{\partial y} - k^2 w\right) - k_0^2 r_0^2\frac{\Delta n(x,y)}{n_0}w. \tag{4}$$

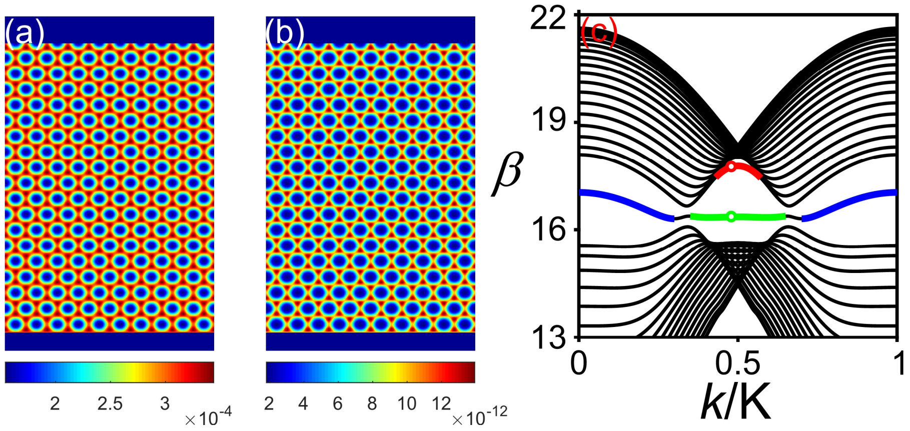

Supplementary Figure 4. (a) The First-order susceptibility. (b) The Third-order susceptibility. (c) Band structure. Parameters are: $N = 3\times10^{12}\ \mathrm{cm}^{-3}$, $\mu_{31} = 1.477\times10^{-29}\ \mathrm{C\cdot m}$, $\gamma_{31} = \gamma_{32} = 2\pi\ \mathrm{MHz}$, and $\gamma_{21} = 0.01\ \mathrm{MHz}$.

By using the plane-wave expansion method and neglecting the nonlinear term in Supplementary

Equation (4), one can obtain the corresponding band structure as shown in Supplementary Fig. 4(c) and edge states as shown in Supplementary Fig. 5. In Supplementary Fig. 4(c), the green curve indicates the edge state on the zigzag edge, while the red and blue curves correspond to the edge states on the bearded edge. Edge states corresponding to the red and green dots in Supplementary Fig. 4(c), where $k = 0.48K$, are shown in Supplementary Figs. 5(a) and 5(b), respectively.

As to the nonlinear edge states, we seek for them in the region where $\beta' > 0$ with small β''. We take the edge state on the zigzag edge as our target. In Supplementary Fig. 5(c), we display corresponding β' (solid curve) and β'' (dashed curve). At $k = 0.48K$, indicated by a dashed line in Supplementary Fig. 5(c), the values of $\beta' \approx 0.034 > 0$ (red dot) and $\beta'' \approx -0.2$ (blue dot) are relatively small.

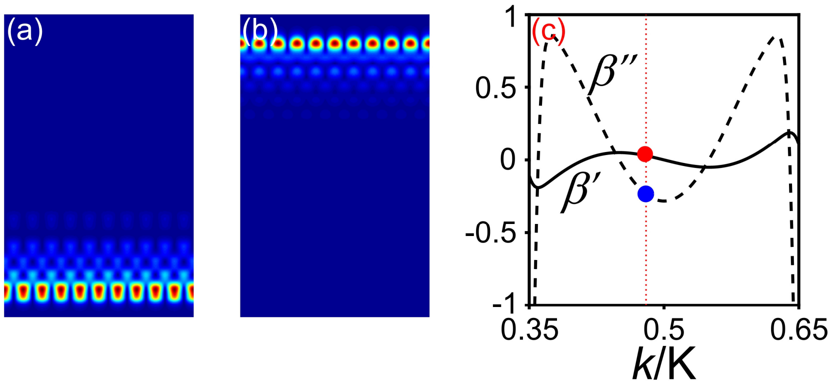

Supplementary Figure 5. (a) Edge state on the bearded edge [red dot in Supplementary Fig. 4(c)]. (b) Edge state on the zigzag edge state [green dot in Supplementary Fig. 4(c)]. (c) $d\beta/dk$ and $d^2\beta/dk^2$ of the green edge state (on the zigzag boundary) in Supplementary Fig. 4. Parameters are same those in Supplementary Fig. 4.

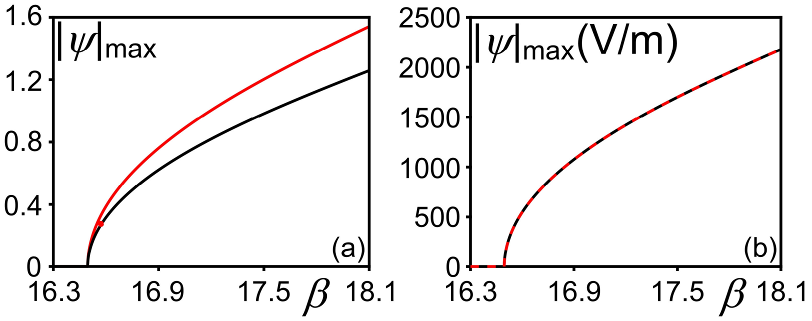

Supplementary Figure 6. (a) Nonlinear edge state amplitude versus β with different scaling factors. (b) Practical nonlinear edge state amplitude versus β.

Since $\chi^{(3)}$ is dimensional, we introduce a dimensional scaling factor s to $\psi \sim s\psi$. As a result, ψ here is also dimensionless, and Supplementary Equation (4) can be rewritten as

$$-w\beta \quad = -\frac{1}{2}\left(\frac{\partial^2 w}{\partial x^2} + \frac{\partial^2 w}{\partial y^2} + 2ik\frac{\partial w}{\partial y} - k^2 w\right) - \frac{k_0^2 r_0^2}{2n_0}\left(\chi^{(1)} + 3s^2\chi^{(3)}|w|^2\right)w. \tag{5}$$

So, now we can use the Newton's method to seek for the nonlinear edge state based on Supplementary Equation (5). In Supplementary Fig. 6(a), we show the amplitude of the nonlinear edge state versus nonlinear energy β. The red and black curves are obtained by setting the scaling factor $s = \sqrt{2} \times 10^3$ mV^{-1}

and $s = \sqrt{3} \times 10^3$ mV^{-1} (which closely correspond to parameters (intensity levels) of the probe beams used in the experiment), respectively. One finds that the nonlinear edge states bifurcate from the linear energy $\beta \approx 16.49$ [shown by the green dot in Supplementary Fig. 4(c)]. We only find the nonlinear edge state in the band gap, so we stop iteration when the energy reaches $\beta \approx 18.1$. In Supplementary Fig. 6(b), we display the real-world amplitude of the nonlinear edge state (with dimension Vm^{-1}) versus β. One finds that the real nonlinear edge state is the same under the two different scaling factors. In Fig. 3 in the main text, we choose $s = \sqrt{3} \times 10^3$ mV^{-1}.

Supplementary Note 5: Propagation dynamics of the edge wave in weakly nonlinear regime

In the absence of the third-order Kerr nonlinearity, the edge states considerably diffract along the edge during propagation [please refer to Fig. 3(g) of the manuscript showing example of linear propagation], but remain confined at the edge. We experimentally tested propagation dynamics of the edge states (with a lower probe power of 100 μW) in the weakly focusing regime, where n_2 is only slightly above 0, as shown in Supplementary Fig. 7. One can clearly see the appearance of the central deep in the pattern and its overall expansion at temperatures exceeding 120°C, but in Fig. 4(b) with $n_2 < 0$ in the main text diffraction occurs at 105°C. So, positive nonlinearity indeed suppresses the diffraction of the edge wave during propagation.

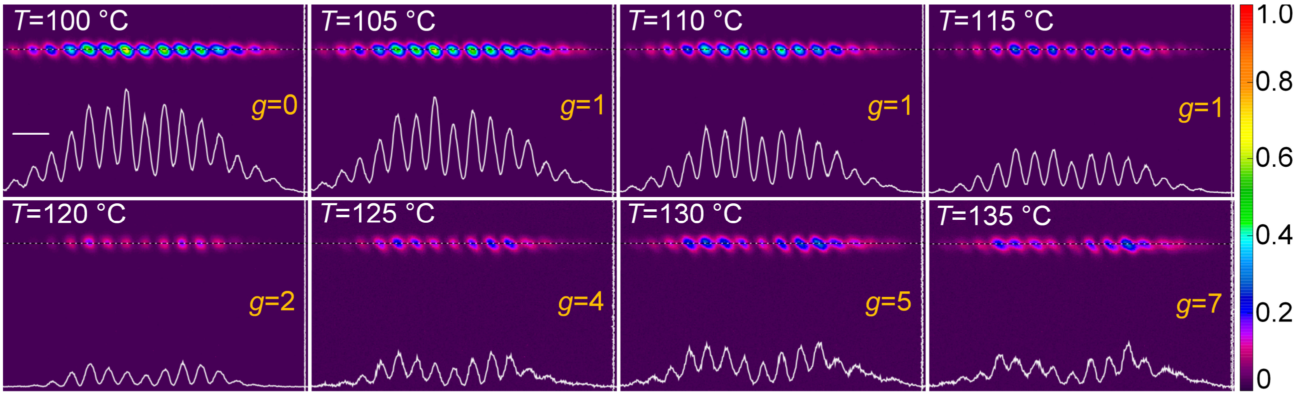

Supplementary Figure 7. Diffraction of the edge state along the edge at n_2 slightly over 0 ($\Delta_1 \approx 133$ MHz) with increase of the temperature. Scale bar: 200 μm.

Supplementary Note 6: Interaction of edge solitons with lattice defects

To illustrate the robustness of the edge solitons in photonic graphene we studied their interactions with lattice defects. The defect was experimentally introduced by injecting a slim Gaussian beam (with the same frequency as the coupling beam and the diameter is ~ 40 μm) from another ECDL to cover one of the waveguides [Supplementary Fig. 8(a)] at the edge. This operation is equivalent to removing one waveguide, and therefore a defect is created on the edge. As shown in Supplementary Fig. 8(b), the edge wave moves along one direction when there is no defect. However, when the defect is introduced, the motion direction of

the edge state is clearly reversed when it meets the defect, while the shape of the state remains practically unchanged [Supplementary Fig. 8(c)]. This is because solitons considered here are not topological, so they may bounce back when they hit the defect upon propagation. We also provide a theoretical illustration of such interaction in Supplementary Fig. 9.

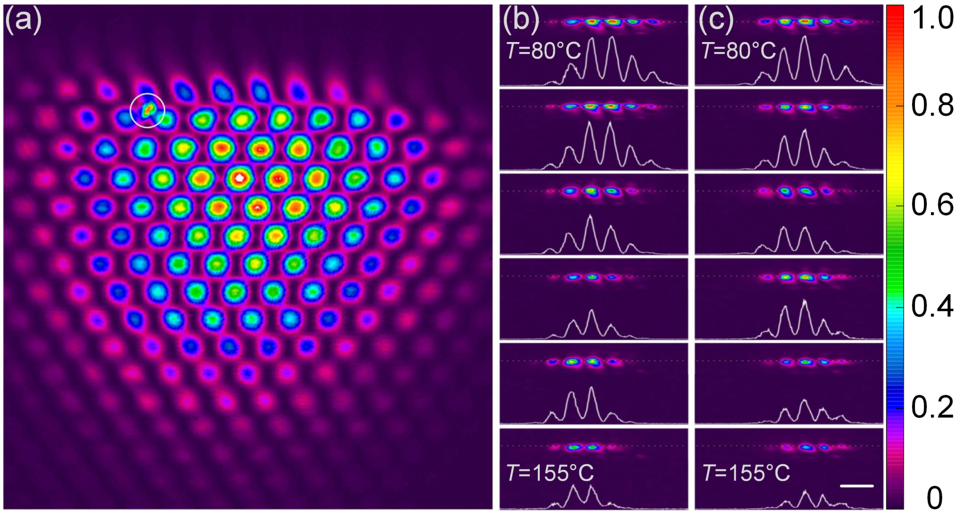

Supplementary Figure 8. (a) Optically-induced defect (marked by the white circle) at the edge. (b) and (c) Propagation of the edge wave without defect (second column) and with the defect (third column), respectively, by increasing the temperature T from 80°C to 155 °C. Scale bar: 200 μm.

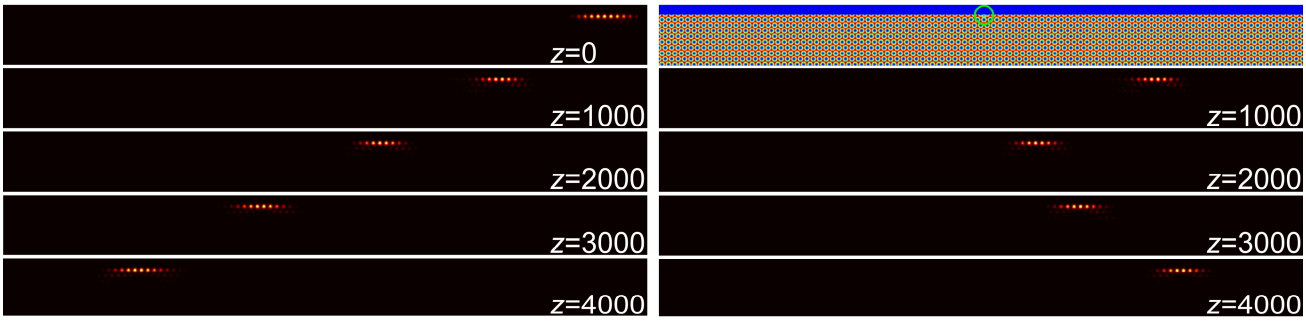

Supplementary Figure 9. Propagation of the edge soliton. Left column: Just as in Fig. 3(f) in the main text, in the absence of a defect the soliton moves along the edge to the left. Right column: We place edge defect (remove one site) at $x = 0$ as indicated by the green circle in the top panel (lattice profile). The soliton, initially moving to the left ($z = 1000, z = 2000$), bounces back on the defect and starts travelling to the right ($z = 3000, z = 4000$).

Supplementary Note 7: Transition from linear diffraction pattern to soliton-like profile by changing input power

To illustrate the impact of the input power of the probe beam on propagation dynamics in the presence of losses, in Supplementary Fig. 10 we show the output edge state profiles for different input powers for the case of $\varDelta_1$=135 MHz and for T=115°C. If the input power P is lower than approximately 200 μW, one can observe clear discrete diffraction with formation of the minimum (indicated by the green arrow) in the center of the output profile, so in this case the probe beam propagates in the effectively linear regime.

However, when the input power exceeds approximately 300 μW, the diffraction is clearly suppressed and one does not observe the dip in the center of the beam anymore. At this frequency detuning (Δ_1=135 MHz), the output intensity distributions exhibit only minor modification (except for growth of the output peak intensity) when the input power further increases from 300 μW to 600 μW. This indicates a clear transition from linear diffraction to the formation of self-sustained states, which indeed dynamically self-adjust their profiles upon propagation through the vapor cell under the unavoidable losses. Such self-adjusting states, closely resembling the states shown in Fig. 4(c) at 400 μW, clearly propagate in the nonlinear regime and even though they cannot be rigorously called solitons (in the sense adopted in conservative systems), they would propagate as solitonic objects if losses are switched off at certain distance.

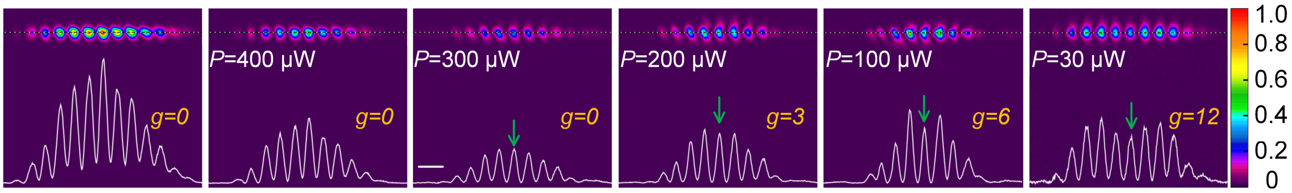

Supplementary Figure 10. Edge states at the output of the cell at different probe powers at Δ_1=135 MHz and T=115°C. Scale bar: 200 μm.

Supplementary Reference

[1] Zhang, Z. *et al.* Observation of parity-time symmetry in optically induced atomic lattices. *Phys. Rev. Lett.* **117,** 123601 (2016).
